# Deletion of the microtubule-associated protein 6 (MAP6) results in skeletal muscle dysfunction

**DOI:** 10.1186/s13395-018-0176-8

**Published:** 2018-09-19

**Authors:** Muriel Sébastien, Benoit Giannesini, Perrine Aubin, Julie Brocard, Mathilde Chivet, Laura Pietrangelo, Simona Boncompagni, Christophe Bosc, Jacques Brocard, John Rendu, Sylvie Gory-Fauré, Annie Andrieux, Anne Fourest-Lieuvin, Julien Fauré, Isabelle Marty

**Affiliations:** 1 INSERM 1216, Grenoble Institute of Neurosciences, F-38000 Grenoble, France; 2 University Grenoble Alpes, F-38000 Grenoble, France; 3 Aix Marseille Univ, CNRS, CRMBM, F-13000 Marseille, France; 4 CeSI-Met & DNICS, University G. d’ Annunzio of Chieti, I-66100 Chieti, Italy; 5 CHU Grenoble, Biochimie et Génétique Moléculaire, F-38000 Grenoble, France; 6 CEA-Grenoble, BIG, F-38000 Grenoble, France; 7 GIN- Inserm U1216 - Bat EJ Safra, Chemin Fortuné Ferrini, 38700 La Tronche, France

**Keywords:** Microtubule-associated protein, Muscle contraction, Calcium release, Microtubules, Sarcoplasmic reticulum, Triad

## Abstract

**Background:**

The skeletal muscle fiber has a specific and precise intracellular organization which is at the basis of an efficient muscle contraction. Microtubules are long known to play a major role in the function and organization of many cells, but in skeletal muscle, the contribution of the microtubule cytoskeleton to the efficiency of contraction has only recently been studied. The microtubule network is dynamic and is regulated by many microtubule-associated proteins (MAPs). In the present study, the role of the MAP6 protein in skeletal muscle organization and function has been studied using the MAP6 knockout mouse line.

**Methods:**

The presence of MAP6 transcripts and proteins was shown in mouse muscle homogenates and primary culture using RT-PCR and western blot. The in vivo evaluation of muscle force of MAP6 knockout (KO) mice was performed on anesthetized animals using electrostimulation coupled to mechanical measurement and multimodal magnetic resonance. The impact of MAP6 deletion on microtubule organization and intracellular structures was studied using immunofluorescent labeling and electron microscopy, and on calcium release for muscle contraction using Fluo-4 calcium imaging on cultured myotubes. Statistical analysis was performed using Student’s *t* test or the Mann-Whitney test.

**Results:**

We demonstrate the presence of MAP6 transcripts and proteins in skeletal muscle. Deletion of MAP6 results in a large number of muscle modifications: muscle weakness associated with slight muscle atrophy, alterations of microtubule network and sarcoplasmic reticulum organization, and reduction in calcium release.

**Conclusion:**

Altogether, our results demonstrate that MAP6 is involved in skeletal muscle function. Its deletion results in alterations in skeletal muscle contraction which contribute to the global deleterious phenotype of the MAP6 KO mice. As MAP6 KO mouse line is a model for schizophrenia, our work points to a possible muscle weakness associated to some forms of schizophrenia.

**Electronic supplementary material:**

The online version of this article (10.1186/s13395-018-0176-8) contains supplementary material, which is available to authorized users.

## Background

Large and highly structured cells such as muscle fibers and neurons have to face considerable challenges to maintain their shape and their different functions after maturation [1, 2]. Many of these functions are sustained by two major elements of the cytoskeleton, the actin filaments, and the microtubules. Muscle’s main function is contraction, which is the outcome of a cascade of events including nerve stimulation and intracellular calcium release, and muscle fiber structure is entirely dedicated to the optimization of contraction. Its cytoplasm is almost fully occupied by the actomyosin filaments of the sarcomeres, and the calcium release required to trigger muscle contraction is performed in specific structures of the muscle, the triads. The actin cytoskeleton was thought to be the major muscle cytoskeletal element, because actin is the main component of the sarcomeres. However, the microtubule cytoskeleton recently gained much attention, as its alteration was shown to contribute to defective muscle function in the Duchenne Muscular Dystrophy [3, 4]. In muscle fibers, the microtubule network forms a dynamic orthogonal grid with longitudinal and transversal microtubule bundles, and is organized as cradles around the multiple peripheral nuclei [5, 6]. Muscle microtubules are required in myoblast fusion and differentiation [7–10], in organelle trafficking [11], and in nuclei positioning [12, 13]. Furthermore, we have recently shown that microtubules are linked to the sarcoplasmic reticulum (SR) and the calcium release complex (CRC) at the triads [14], pointing to their possible involvement in calcium release for muscle contraction.

Until now, only a few microtubule-associated proteins (MAPs) such as MAP7 or oMAP4 have been characterized as being involved in muscle differentiation and functions [8, 13]. MAP6, previously called STOP protein, has been isolated from brain preparations [15, 16] but is also present in other tissues such as the skeletal muscles and heart [17]. Several isoforms are produced from the alternative splicing of the *MAP6* gene, the major ones being MAP6-N, MAP6-F, and MAP6-E corresponding respectively to the neuronal, fibroblastic, and embryonic isoforms, with additional poorly characterized minor isoforms [18, 19]. MAP6 isoforms are known to stabilize microtubules in vitro against cold temperature, depolymerizing drugs like nocodazole, and high calcium concentrations [18, 20]. MAP6-N and MAP6-E are associated in neurons with cold-stable, drug-resistant, and long-lived microtubules [21]. MAP6-F exhibits different locations in fibroblasts depending on the temperature: at 37 °C, it has a diffuse pattern in the cytoplasm, whereas at 4 °C MAP6-F associates with and stabilizes microtubule arrays [19, 22]. In neurons, MAP6 proteins exhibit other functions than those related to microtubules. First, they are able to interact with the actin cytoskeleton [23]. Second, thanks to reversible palmitoylation at the N-terminal end, the neuronal isoforms can be targeted to different locations (plasma membrane, Golgi apparatus, and mitochondria) [24, 25], and this dynamic palmitoylation may favor neuronal polarization [26]. Finally, MAP6 exhibits a signaling function independent of microtubule binding, promoting axonal attractive guidance downstream of semaphorin 3E [27]. Hence, MAP6 proteins are now considered as scaffold proteins which can integrate multiple cellular roles ranging from cell signaling to cytoskeleton stabilization. Reflecting the multiple functions of MAP6 proteins at the cellular level, MAP6 null mice (MAP6 KO), which are devoid of all MAP6 isoforms, are viable but display severe behavioral disorders resembling schizophrenia-related symptoms [28–31]. Indeed, treatment with anti-psychotic drugs alleviates several of the behavioral and biological defects [32–34].

The diverse functions of MAP6 have until now been extensively studied in the brain but never in other differentiated tissues such as skeletal muscle. In this work, we show the presence of MAP6 isoforms in skeletal muscle, and we show that MAP6 KO mice exhibit muscle weakness together with muscle atrophy. The structure and function of the muscle fibers of MAP6 KO mice show several alterations in intracellular organization as well as in the calcium release mechanism. Altogether, our study points to the importance of MAP6 proteins in muscle function.

## Methods

### Antibodies

A primary antibody directed against a central repeat motif of MAP6 proteins (antibody 23N), described previously [19], was used to label all the MAP6 isoforms. The antibody against the alpha-1 subunit of dihydropyridine receptor (DHPR) was from Abcam (#Ab2862), the antibodies against β-tubulin (TUB2.1, #T5201) and alpha-actinin (A-7811) were from Sigma, and the antibody against Golgi apparatus was from Santa Cruz (FL-145). Antibodies against RyR1, triadin T95, tyrosinated tubulin (YL1/2), and detyrosinated tubulin were previously described [35–37]. The antibody against SERCA was kindly provided by Dr. M.-J. Moutin [38].

### Animals

MAP6 KO mice were developed by insertion of LacZ gene instead of the exon 1 of *MAP6* gene leading to the extinction of all MAP6 protein isoforms as described before [28]. The MAP6 KO and wildtype (WT) littermate mice on homogeneous background C57BL6/129svPas-F1 were obtained by crossing heterozygote animals (MAP6 pure heterozygote 129svPas male or female with MAP6 pure heterozygote C57BL6 male or female mice). Mice from 3 to 10 months old were considered as adults. Mice breeding was performed in compliance with the French legislation and European Union Directive of 22 September 2010 (2010/63/UE). All procedures using animals were approved by the Institutional ethics committee (C2EA-04) and followed the guidelines of the National Research Council Guide for the care and use of laboratory animals.

### Western blot analysis

Muscle (quadriceps) or brain homogenates were prepared as described previously from frozen tissues [14]. The presence and the amount of different proteins in tissue homogenates was assayed by Western blot analysis, using a chemiluminescent reagent after electrophoretic separation of the protein on a 5–15% acrylamide gel, and electrotransfer on Immobilon P (Millipore) [39]. The quantification was performed using a ChemiDoc Touch apparatus (Biorad, Marnes-la-Coquette, France) and the ImageLab Software (Biorad). The signal was normalized to the amount of a muscle-specific protein of low abundance (triadin T95) or GAPDH.

### Histological staining

Tibialis anterior was collected immediately after euthanasia, flash-frozen in liquid nitrogen, and embedded in optimal cutting temperature (OCT) compound (Tissue Tek, Sakura). Transversal cryosections (10 μm) were processed for hematoxylin and eosin (H/E) and nicotinamide adenine dinucleotide (NADH) staining. Images were acquired using Axioscan Z.1 (Zeiss) and cross-sectional area (CSA) and analysis was performed using ImageJ software. CSA fibers from three different animals were analyzed for each genotype (WT CTR: 412 fibers, KO MAP6: 459 fibers), and the values are presented as mean ± SEM.

### Cell culture

Primary muscle cultures were prepared from hind limbs of newborn WT and MAP6 KO mice, as described previously [40], and expanded in proliferation medium Ham’s F-10 (Life technologies, Saint Aubin, France) supplemented with 20% FBS (Life technologies, Saint Aubin, France), 2% Ultroser G (Pall Biosepra, St Germain en Laye, France), and 2% Penicillin-Streptomycin (Life technologies, Saint Aubin, France). C2C12 (ATCC ref. CRL-1772) were amplified in DMEM (Life technologies, Saint Aubin, France) supplemented with 10% fetal bovine serum (Life technologies) and 1% penicillin-streptomycin (Life technologies). Differentiation into myotubes was induced by a shift to differentiation medium: DMEM 1 g/L (Life technologies, Saint Aubin, France) supplemented with 2% Heat Inactivated Horse Serum (Life technologies) and 1% Penicillin-Streptomycin.

### Calcium imaging

Primary muscle cells from WT and MAP6 KO were seeded on laminin-coated culture dishes. After 3 days of differentiation, changes in intracellular calcium were measured on WT or KO myotubes, using the calcium-sensitive fluorescent dye Fluo4 direct (Thermo Fisher Scientific). Stimulations were performed in Krebs buffer (136 mM NaCl, 5 mM KCl, 2 mM CaCl_2_, 1 mM MgCl_2_, 10 mM HEPES, pH = 7.4) with 500 μM 4-Chloro-m-cresol (4CmC) or 140 mM KCl, and fluorescent variations were recorded on a Leica SPE confocal microscope and analyzed as described previously [39].

### RNA extraction and RT-PCR

Brains and muscles from WT mice were collected and frozen in liquid nitrogen. Tissues or C2C12 myotubes were homogenized in Trizol (Thermo Fisher Scientific) according to manufacturer procedure, and mRNA was purified on Purelink RNA columns (Life technologies). After reverse transcription using oligo-dT primer, PCR amplification was performed with Phusion master Mix (Thermo Scientific) using the following procedure: after an initial denaturation of 1 min at 98 °C, 30 cycles of PCR amplification (10 s at 98 °C, 30 s at 69 °C, 15 s at 72 °C) were performed, using the following primers for the amplification of the different MAP6 exons, designed in non-repeated sequences of the four exons of MAP6: exon 1 forward (5′-GAGGAGGTGGCGAGTACAGT-3′), exon 2 forward (5′-CCCCAGATGACAAGATGGTT-3′), exon 3 reverse (5′-TTCGCCTCAGCCAGTTTATT-3′), exon 4 reverse (5′-GATGCATCACTGGTGGGTTT-3′).

### RT-q-PCR

RNA extracted was reverse transcribed using the iScript Reverse Transcription Supermix (Bio-Rad) following the manufacturer’s instructions. Gene expression was measured by RT-qPCR using the SsoAdvanced Universal Sybr green supermix (Bio-Rad) and the C1000 Touch Thermal Cycler–CFX96 Real-Time System (Bio-Rad). The list of specific primers (Eurofins) is provided in Table 1.

**Table 1:** Primers for RT-q-PCR amplifications

Mouse gene	Forward (5′–3′)	Reverse (5′–3′)
β-actin	GACAGGATGCAGAAGGAGATTACTG	CTCAGGAGGAGCAATGATCTTGAT
Chrng	AGCCTCCCCAGCCATCCAGG	GGCCCACCAGCAACCACTCC
Map6_ex1-2	CCCTCAACAGGCAAATCC	TCTCATGAACCATCTTGTCATC
Musk	ATCACCACGCCTCTTGAAAC	TGTCTTCCACGCTCAGAATG
Myogenin	CTTGCTCAGCTCCCTCAAC	TGGGAGTTGCATTCACTGG
Runx1	GAAGAACCAGGTAGCGAGATTC	GTAAAGACGGTGATGGTCAGAG

### cDNA ends amplification

>cDNA 5′ ends amplifications were performed using the SMARTer RACE kit (Clontech), according to manufacturer protocol, with the following primer in MAP6 exon 2: 5′-GATTACGCCAAGCTTCTTCGGGCATTCCTTGAAAGGCTCACT G-3′. The major PCR products were purified with a PCR clean up kit (Macherey-Nagel) and sequenced.

### Muscle fiber isolation and immunolabeling

Isolated muscle fibers were prepared by enzymatic and manual dissociation of *flexor digitorum brevis* (FDB) muscles, and the immunofluorescent labeling after fixation was performed as described previously [14]. Images were acquired with a 63× objective on an LSM710 confocal microscope (Zeiss), equipped with an Airy-scan module. To quantify the global microtubule density, binary masks corresponding to longitudinal or transversal microtubule areas were generated with a homemade ImageJ macro. The obtained densities were corrected with XLSTAT software for day-dependent parameters, and mean values were calculated and represented with Graphpad Prism 6.0 software. For a more detailed analysis of microtubule network organization along the fiber, the number of microtubule crossing a longitudinal line and the intensity profile along this line were recorded and normalized to the highest peak. Forty different fibers from three different animals were analyzed for each genotype, and the values are presented as mean ± SEM.

### In vivo force measurements

Mechanical performance measurements coupled to multimodal MR data acquisition were done as previously described on the left *gastrocnemius* muscle of anesthetized mice (6 WT and 7 MAP6 KO male mice, 7 to 8 months old) using a totally non-invasive procedure [39, 41]. Throughout each experiment, general anesthesia was ensured by isoflurane inhalation, and animal body temperature was controlled and maintained at physiological value. Muscle contractions were induced by transcutaneous electrostimulation, and mechanical performance (i.e., force-generating capacity) was evaluated during a fatiguing bout of exercise consisting in 6 min of repeated maximal isometric contractions induced at a frequency of 1.7 Hz. Specific twitch tension was determined by normalizing absolute twitch tension to muscle volume calculated from hindlimb MR imaging.

### Electron microscopy

*Extensor digitorum longus* (EDL) muscles were dissected from 4 months old animals (3 WT and 3 MAP6 KO mice) at resting length and prepared for standard and transverse-tubules staining electron microscopy as described in [42]. Specimens were cut in ultrathin sections using a Leica Ultracut R microtome (Leica Microsystem) with a Diatome diamond knife (Diatome Ltd.). Ultrathin sections (~ 50 nm) were examined after staining in 4% uranyl acetate and lead citrate, with a Morgagni Series 268D electron microscope (FEI Company), equipped with Megaview III digital camera.

### Quantitative analysis by electron microscopy

For all quantitative analyses, micrographs of non-overlapping regions were randomly collected from transversal and longitudinal sections of internal fiber areas. The percentage of fibers exhibiting stacks of flat SR membranes and their number per 100 μm^2^ area/section were evaluated at 28,000 magnification in 5 micrographs from 10 fibers of each specimen. The relative fiber volumes occupied by free SR and free SR surface area to fiber volume ratio were measured in transversal sections by using the well-established stereology point and intersection counting method [43, 44]. In each specimen, 10 fibers were analyzed and in each fiber 1–2 micrographs were taken at 22,000 magnification. Number of triads per area was evaluated in micrographs from longitudinal sections and was reported as average number over 100 μm^2^ (see [42] for more detail). In each electron microscopy (EM) micrograph, the orientation (oblique or longitudinal) of triads has been determined. In each specimen, 10 fibers were analyzed and in each fiber 6 micrographs were taken at 14,000 magnification.

### Statistical analysis

The statistical analyses were performed using Student’s *t* test or the Mann-Whitney test according to samples amount and distribution.

## Results

### MAP6 is present in skeletal muscle

**Fig. 1:**
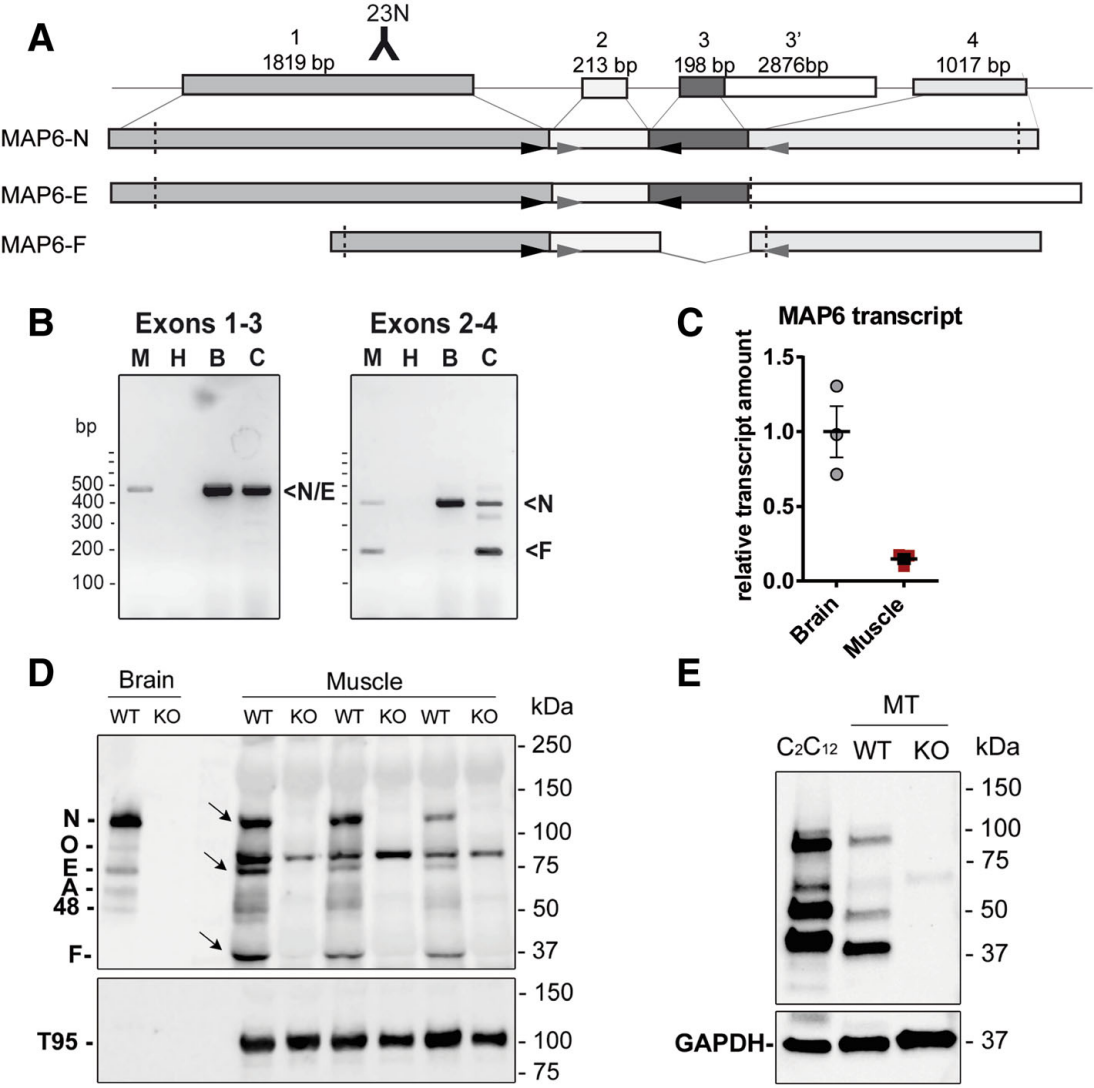
MAP6 isoforms are present in mice skeletal muscle. **a** Structure of the *MAP6* gene and of the major mRNA isoforms obtained by the alternative splicing of the four exons or the use of an alternative promoter in the case of MAP6-F. The dashed lines represented for each isoform, the localization of the start, and the stop codons. The localization of the antigen for the 23N antibody is represented. The localization of the primers used to amplify the four exons is represented by the arrowheads. **b** RT-PCR amplifications from muscle (M), brain (B), or C2C12 myotube (C) extracts, with H_2_O (H) instead of cDNA as a negative control. PCR fragments at sizes corresponding to MAP6-N, E and F transcripts were amplified from brain homogenates: 443 bp for exons 1–3 (N/E), and 373 bp (major-N) and 176 bp (minor-F) for exons 2–4. In muscle homogenates and in C2C12, PCR fragments of the same size as in brain homogenates were amplified. **c** RT-q-PCR amplification of MAP6 transcripts in brain or muscle extracts, from three different mice, compared to β-actin. **d** Western blot analysis has been performed with antibody 23N on 60 ng whole brain homogenates prepared from WT or MAP6-KO animals, and 50 μg of muscle homogenate prepared from WT or MAP6-KO skeletal muscle (three mice of each genotype). In brain, five proteins are detected, the N, O, E, A, and an un-characterized 48-kDa isoform (48). The three bands specifically detected in WT skeletal muscle at 130 kDa (N-isoform), 75 kDa (E-isoform), and 42 kDa (F-isoform) are marked with arrows. The muscle-specific triadin isoform Trisk 95 (T95) has been used as a loading control (lower panel). **e** Western blot analysis has been performed with antibody 23N on C2C12 homogenate or 3 days WT and MAP6-KO myotube (MT) homogenates. GAPDH has been used as a loading control (lower panel)

MAP6 transcripts and proteins have been detected in skeletal muscle, but without detailed characterization of the expressed isoforms [17]. We used RT-PCR amplification to identify the transcripts expressed in skeletal muscle. The major neuronal isoform MAP6-N contains exons 1 to 4, while the so-called embryonic isoform MAP6-E contains exons 1, 2, 3, 3′ and the fibroblastic isoform MAP6-F contains exons 1, 2, and 4 with the use of an alternative promoter in exon 1 (Fig. 1a). Using different combinations of primers, RT-PCR was performed on wildtype (WT) skeletal muscle and brain homogenates, and on extracts produced from differentiated C2C12 myotubes, a mouse muscle cell line used as a control (Fig. 1b). Amplification of exons 1 to 3 showed a single band for muscle (M), brain (B), and C2C12 (C) that could correspond to MAP6-N and MAP6-E transcripts. Amplification of exons 2 to 4 revealed two bands: one that could correspond to MAP6-N transcript and one that could correspond to MAP6-F transcript (Fig. 1b). This demonstrates that MAP6 transcripts are present in mouse skeletal muscle, although at different expression levels than in the brain (Fig. 1b). The similar pattern observed in whole muscle and in a pure myotube population from C2C12 culture confirmed the presence of MAP6 transcripts in muscle cells, excluding any possible contamination by another cell type (peripheral neurons or fibroblasts for instance). The relative MAP6 transcripts levels in brain and muscle tissues were further determined by RT-q-PCR (Fig. 1c), and a sevenfold reduction was observed in muscle compared to the brain. Amplification of the 5′-cDNA end from a primer in the common exon 2, followed by partial sequencing of the major bands confirmed that N/E and F transcripts are present in skeletal muscle (Additional file 1: Figure S1). We further studied the presence of the proteins using western blot with an antibody directed against a peptide localized in a common region of exon 1 (Fig. 1d), and triadin, a muscle-specific protein of moderate abundance, as a loading control. Three bands were specifically detected in control muscle compared to MAP6 KO muscle, corresponding to the MAP6-N, MAP6-E, and MAP6-F isoforms, with possibly additional ones in minor amounts (Fig. 1d) [45]. The presence of MAP6 proteins was confirmed in the pure myoblast cell line (C2C12, Fig. 1e), and in differentiated myotubes (WT compared to MAP6 KO myotubes Fig. 1e). The expression pattern, similar between the two muscle cell types, is different from adult muscle. The non-specific bands recognized by this antibody in skeletal muscle precluded the further characterization of these isoforms and the study of their localization by immunofluorescent labeling, and indeed the same labelling was observed on WT and MAP6 KO muscles.

### MAP6 KO muscle does not show signs of denervation

**Fig. 2:**
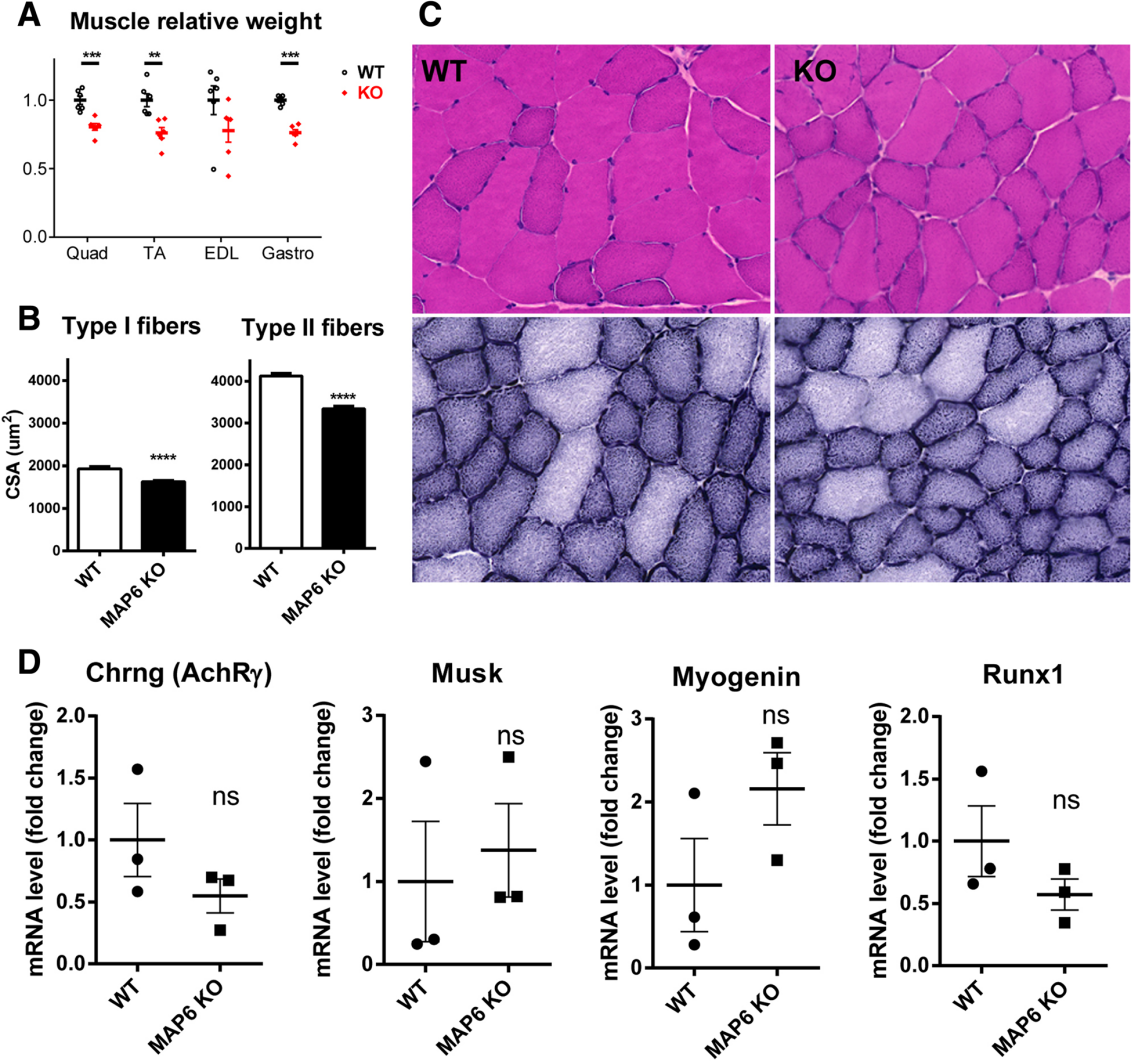
Characterization of MAP6 KO skeletal muscles. **a** The weight of different muscles was measured from three WT and three MAP6 KO mice and normalized to weight of WT muscles. **b** The cross-sectional area (CSA) was measured in type I and type II fibers from WT and MAP6 KO *tibialis anterior*. **c** Hematoxylin/eosin staining (upper panels) and NADH staining (lower panels) of WT and MAP6 KO muscles. **d** Quantitative RT-PCR amplification of four genes markers of denervation: AChR-γ; Musk; Myogenin; Runx1

The global muscle structure was analyzed using histological staining. The weight of different muscles was measured and a reduction of about 20% compared to WT was observed in all the MAP6 KO muscles tested (Fig. 2a). At the fiber level, a global reduction of about 20% in the size of all fibers (16% for type I fibers and 19% for type II fibers) has also been measured (Fig. 2b). Using H&E and NADH stainings, no gross modification was observed: no regenerating fibers, central nuclei, nor fibrosis (Fig. 2c). No histological sign of denervation was observed, which was confirmed by following the expression of a set of genes known to be upregulated in the case of denervation (AChR-γ; Musk; Myogenin; Runx1) [46]. RT-q-PCR amplification of these genes showed that their expression was not significantly increased in MAP6 KO muscle compared to WT (Fig. 2d). Therefore, the defects observed in MAP6 KO muscles and the muscle atrophy are most probably mainly related to intrinsic muscle defects and not subsequent to muscle denervation.

### MAP6 KO mice show muscle function defects in vivo

**Fig. 3:**
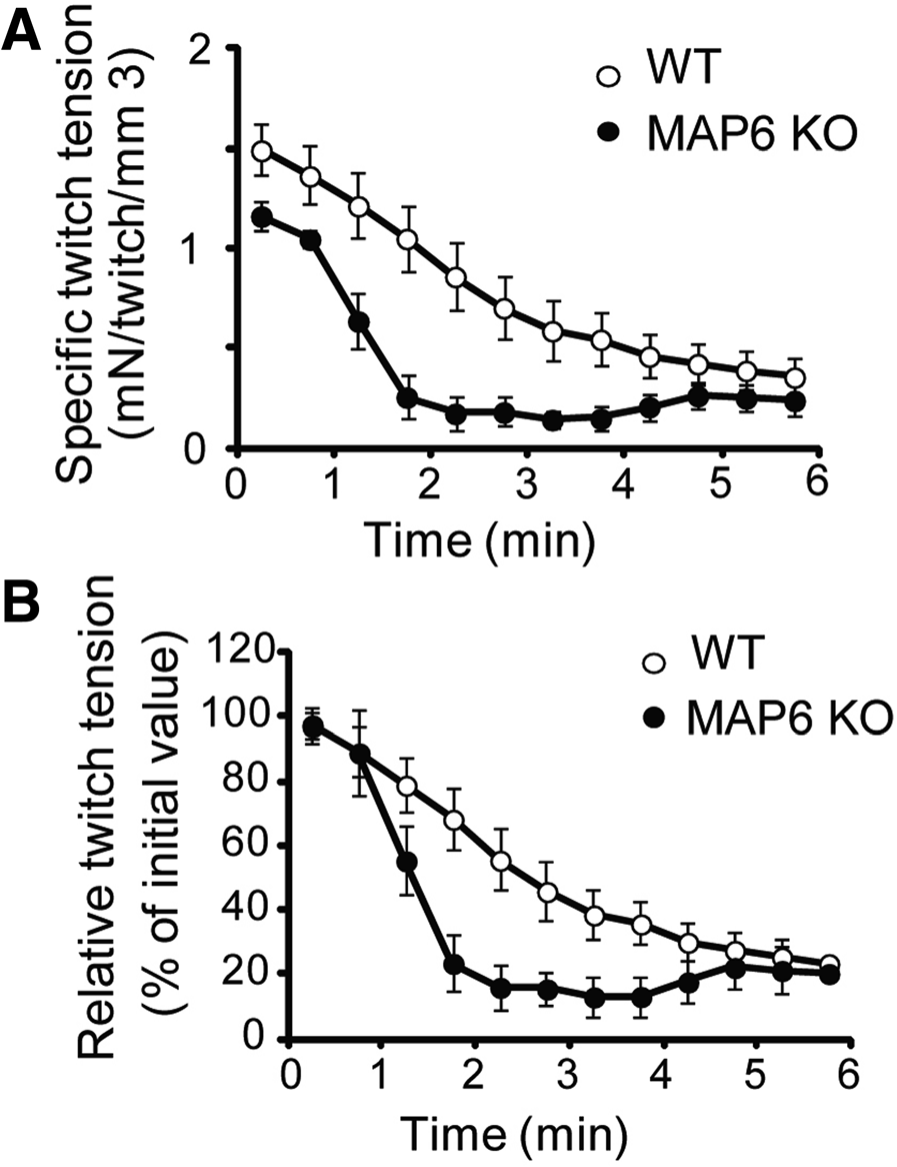
*Gastrocnemius* muscle mechanical performance is reduced in MAP6 KO mice. Changes in specific (**a**) and relative (**b**) twitch tension throughout a 6-min in vivo fatiguing bout of exercise performed simultaneously to bioenergetics MR acquisition. Data are means ± SEM on six WT and seven KO mice

Muscle function was further studied in the MAP6 KO mouse line. Due to the multiple behavioral defects of these mice, like an acute response to stress [28–30], the use of classical force measurement tests (treadmill run or grip tests) was not possible. Therefore, we used a non-invasive protocol involving transcutaneous electrostimulation coupled to mechanical measurement and multimodal magnetic resonance (MR) acquisition to evaluate *gastrocnemius* muscle function in anesthetized animals [41]. Because the absence of MAP6 results in synaptic function alteration [32, 47], muscle electrostimulation was performed by a direct depolarization of the plasma membrane, bypassing nerve stimulation. The muscle volume and the twitch tension developed during a 6-min fatiguing bout of exercise were measured in 8 months old male mice (Fig. 3a, b and Table 2).

**Table 2:** Body weight, *gastrocnemius* muscle volume, and in vivo mechanical performance

	WT	MAP6 KO
Body weight (g)	39.3 ± 2.6	35.1 ± 2.0
*Gastrocnemius* volume (mm^3^)	*142 ± 3*	*128 ± 2* *
Maximal specific twitch tension (mN/twitch/mm^3^)	*1.53 ± 0.12*	*1.19 ± 0.07* *
End exercise twitch tension (% of start exercise value)	24 ± 6	20 ± 7
Total tension produced during the whole 6-min exercise (mN/mm^3^)	*440 ± 70*	*210 ± 30* *

*Gastrocnemius* volume was calculated in vivo from MR images acquired at rest on anesthetized animals, and mechanical performance was assessed during the 6-min fatiguing exercise for six WT and seven MAP6 KO male mice. Data are presented as mean ± SEM. The Mann-Whitney tests were performed and significant differences (*p < 0.05) are italicized

The animals presented similar average body weights, but the MAP6 KO *gastrocnemius* volume calculated from hindlimb MR imaging was reduced compared to WT (from 142 ± 3 mm^3^ in WT to 128 ± 2 mm^3^ in KO) (Table 2), pointing to a small yet significant muscle atrophy (~ 10% muscle volume reduction), confirming the muscle atrophy previously observed at the muscle and fiber levels (Fig. 2). The muscle capacity for generating specific twitch tension (i.e., absolute twitch tension normalized to muscle volume) was dramatically reduced in MAP6 KO animals whereas the extent of twitch tension reduction at the end of exercise did not differ between both phenotypes (Table 2 and Fig. 3). Interestingly, there were no differences between WT and KO muscles for intracellular pH, adenosine triphosphate (ATP), and PCr (phosphocreatine) levels, monitored dynamically using ^31^P-MR throughout rest, exercise, and recovery periods (Additional file 2: Figure S2 and Additional file 3: Table S1), which indicates that basal bioenergetics status and energy demand during exercise were not disturbed in mice lacking MAP6, as already observed on another study on the same model [48]. These data demonstrate that the absence of the microtubule-associated protein MAP6 induces a muscle weakness that was not accompanied by a proportional reduction in energy demand, hence demonstrating an impairment of the contractile energetic efficiency.

### MAP6 deletion in mice affects microtubule organization

**Fig. 4:**
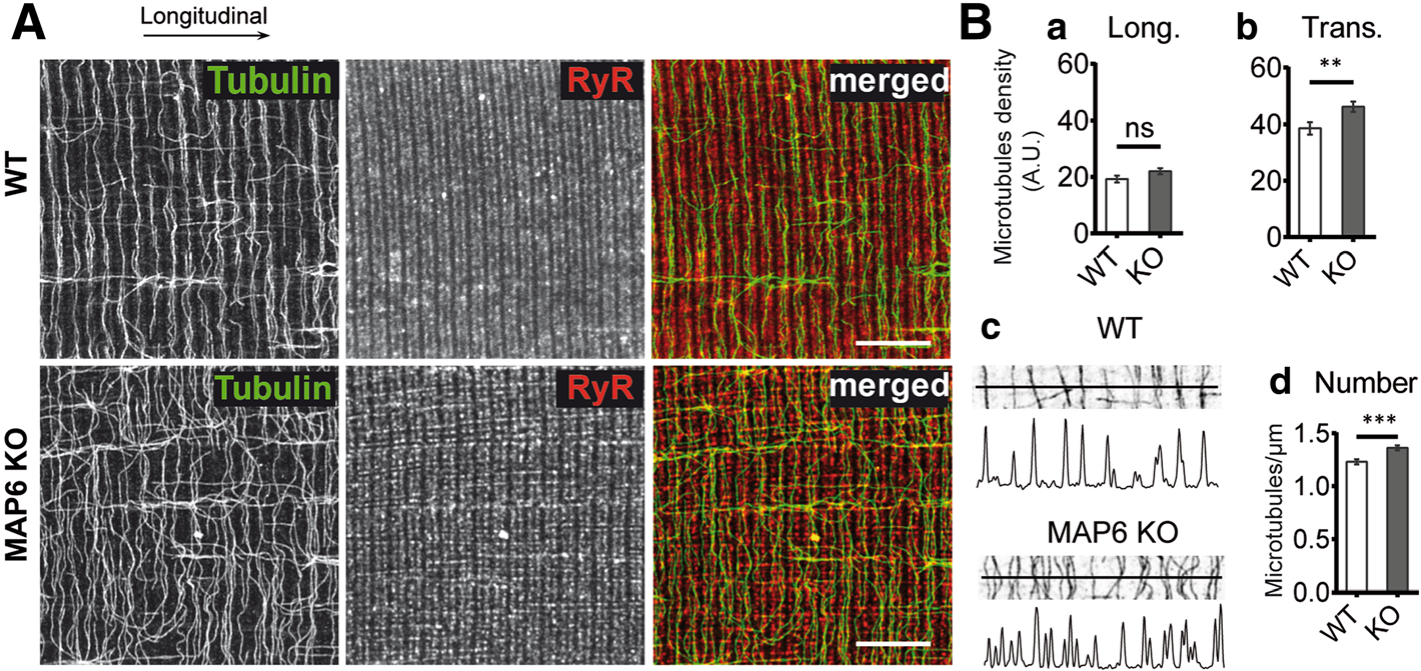
Transversal microtubules organization differs between WT and MAP6 KO fibers. **A** FDB muscle fibers dissociated from WT and MAP6 KO adult mice were fixed at 37 °C before labeling with anti-β-tubulin (green) and anti-RyR1 (red) antibodies. Each image represents a single confocal plane. Scale bars: 10 μm. **B** The microtubule network density was assessed on 40 WT and 40 KO fibers depending on its orientation: either longitudinally oriented (**a**) or transversally oriented (**b**) compared to the fiber longitudinal axis. To get more details on microtubule network organization, longitudinal lines were drawn on each cell (**c**), and the intensity profile was recorded for each, allowing the determination of the number of peaks per micrometer for WT and MAP6 KO cells (**d**), which correspond to the number of transversal microtubules per micrometer. Values are represented as means ± SEM, Student’s *t* test, ***p < 0.001, **p < 0.01 and ns: non-significant

We next investigated the molecular mechanisms linking MAP6 deletion to muscle weakness. As MAP6 function has been related to microtubule stability, the absence of MAP6 protein could therefore lead to microtubule network destabilization and muscle intracellular disorganization. We thus studied the microtubule cytoskeleton and the general muscle subcellular organization in isolated FDB muscle fibers from WT and MAP6 KO mice, using immuno-fluorescent staining of β-tubulin to label microtubules, with a co-staining of the calcium channel-ryanodine receptor (RyR1) as a triad marker and of alpha-actinin (a Z-line protein) as a sarcomere marker (Fig. 4 and Additional file 4: Figure S3). For both genotypes, triads and sarcomeres had a normal organization, with double rows of triads located on both side of the Z-line of the sarcomeres (Additional file 4: Figure S3). Microtubules exhibited a grid-like network similar to the one described in several studies [5, 14, 49]; however, it seems organized differently between WT and MAP6 KO muscle fibers (Fig. 4A). To assess these differences, quantifications of the longitudinal and transversal microtubule density as well as the average number of transversal microtubules crossing a longitudinal line, were performed (Fig. 4B). Surprisingly, the transversal microtubule density was higher in MAP6 KO fibers than in WT (Fig. 4B, b), and the average number of transversal microtubule crossing a longitudinal line was slightly but significantly increased in MAP6 KO (from 1.23 ± 0.02/μm in WT fibers to 1.36 ± 0.02/μm in MAP6 KO fibers (Fig. 4B, c, d). These results suggest that there are more transversal microtubules in MAP6 KO than in WT fibers or that the transversal microtubules are less bundled in the absence of MAP6. Quantitative western blot analysis of the total amount of tubulin in WT and MAP6 KO muscles showed no difference (Additional file 5: Figure S4). Tubulin post-translational modifications, especially detyrosination, have been involved in contraction efficiency [50, 51]. Therefore, we checked the relative amount of tyrosinated/detyrosinated microtubules in WT and MAP6 KO muscles (Additional file 6: Figure S5) and did not observe any difference. Overall, our results favor the hypothesis of a change in microtubule organization rather than in microtubule quantity or tubulin post-translational modification.

### MAP6 KO muscles present SR structural abnormalities

**Fig. 5:**
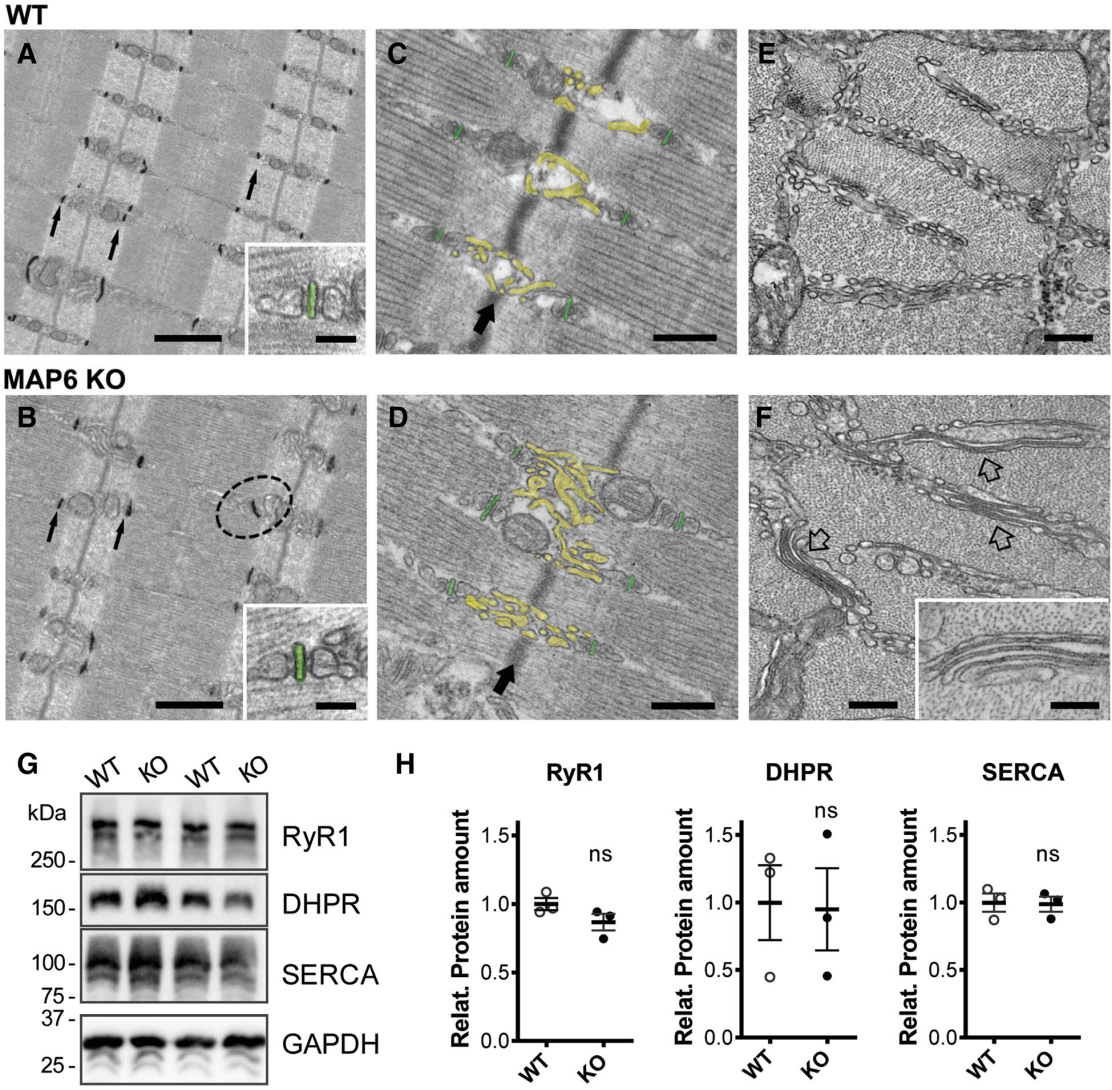
EM reveals a remodeling of the sarcoplasmic reticulum at the I-band in MAP6 KO fibers. **a**, **b** EM images of EDL fibers with transverse tubule (TT) staining (dark). In the insets, representative images of triads with TT pseudo-colored in green. Dotted circle in **b** points to an obliquely oriented TT, instead of the normal transversal TT orientation (thin black arrows). **c**, **d** EM pictures showing the organization of SR membranes at the I-band. Large arrows point to Z-lines. The free SR is colored in yellow and transverse tubules in green. **e**, **f** EM images of muscle cross sections. Empty arrows point to stacks of flat and parallel SR cisternae (one of them being enlarged in the inset). All images are representative of at least 30 fibers from 3 different animals of each genotype. Scale bars: **a**–**b**, 1 μm (inset: 0.05 μm); **c**–**d**, 0.2 μm; **e**–**f**, 0.1 μm (inset: 0.05 μm). **g** Representative western blots on two different mice of each genotype. **h** Quantitative analysis of CRC protein amounts: RyR1, alpha-1-subunit of DHPR, and SERCA in skeletal muscle homogenates. The amount of protein was normalized to GAPDH expression, the WT mean values being set to 1. *n* = 7 blots from three WT and three MAP6 KO mice, Mann-Whitney, ns: non-significant

Whereas the general fiber organization seemed not impaired at the scale of immunofluorescent analysis, a detailed ultrastructural analysis using electron microscopy (EM) was performed to visualize more subtle modifications in MAP6 KO muscles that could contribute to the resulting muscle weakness [39, 52, 53]. In WT muscle fibers, as classically observed, calcium release units were located at the sarcomere A-I junctions and were mostly in the form of triads: two SR vesicles apposed to a central transverse-tubule (TT), which are oriented transversally with respect to the long axis of myofibrils (Fig. 5a). In most of the MAP6 KO fibers analyzed, the overall fiber ultrastructure was well preserved: myofibrils were aligned with one another and triads and TTs were correctly disposed (Fig. 5b). Quantitative analysis confirmed indeed that there was no difference in the density of triads (Additional file 7: Table S2, line A) and in the percentage of longitudinally oriented triads, i.e., triads in which TTs were oriented longitudinally with respect to the long axis of myofibrils (Additional file 7: Table S2, line B). Only a significant increase in the percentage of oblique triads (circled in Fig. 5b) was detected (Additional file 7: Table S2, line C), i.e., triads in which TTs were not perfectly transversally oriented (1.7 ± 0.4% in WT vs 5.6 ± 0.8%, in MAP6 KO). However, more striking differences between WT and MAP6 KO EDL fibers were detected when analyzing the ultrastructure of the free SR (or longitudinal SR) at the I-band level between triads (Fig. 5c–f). In WT fibers, the free SR at the I-band on both sides of Z lines was composed of convoluted tubules that, when cross-sectioned, appeared as multiple layers of vesicles (Fig. 5e). In MAP6 KO fibers, the free SR at the I-band appeared remodeled to form straighter tubes and/or flat cisternae (Fig. 5d), which often were stacked in multiple layers (pointed by arrows in Fig. 5f and enlarged in the inset). The number of fibers presenting stacks and the number of stacks per area increased significantly in EDL fibers from MAP6 KO mice (Table 3, lines A and B). After quantitative analysis of free SR membranes (Table 3, lines C and D), a small but significant increase in both SR volume and surface in MAP6 KO fibers compared to WT was confirmed. These results showed that localization and structure of triads were very mildly affected by deletion of MAP6 proteins and suggest that deletion of MAP6 impacted mostly the morphology of the SR at the I-band.

**Table 3:** EM analysis of EDL from WT and MAP6 KO

		WT	KO
A	Fibers with SR membrane stacks (% of total)	23.5 ± 3.5	64.0 ± 4.4**
B	N. of SR stacks /100 μm^2^	3.4 ± 0.6	10.6 ± 0.9**
C	Free SR volume/total volume (%)	5.3 ± 0.4	6.5 ± 0.3**
D	Free SR surface area/total volume (μm^2^/μm^3^)	1.2 ± 0.1	1.5 ± 0.1*

Data are shown as mean ± SEM. Student’s *t* test, *p < 0.05 and **p < 0.01 vs WT. Sample size: rows A and B: 20–30 fibers, 2 micrographs/fiber (22,000 magnification images in transversal sections); rows C and D: 30–45 fibers; 5 micrographs/fiber (28,000 magnification images in transversal sections)

One major function of SR in muscle is calcium storage for contraction; therefore, the amounts of the main proteins involved in calcium homeostasis were evaluated using quantitative western blot in WT and MAP6 KO muscle homogenates (Fig. 5g, h). No significant difference was observed for the tested proteins (RyR1, the alpha1-subunit of DHPR and the Ca^2+^-ATPase SERCA), pointing to a remodeling of SR membranes rather than a modification of their quantity or composition.

### Calcium release is reduced in MAP6 KO cells

**Fig. 6:**
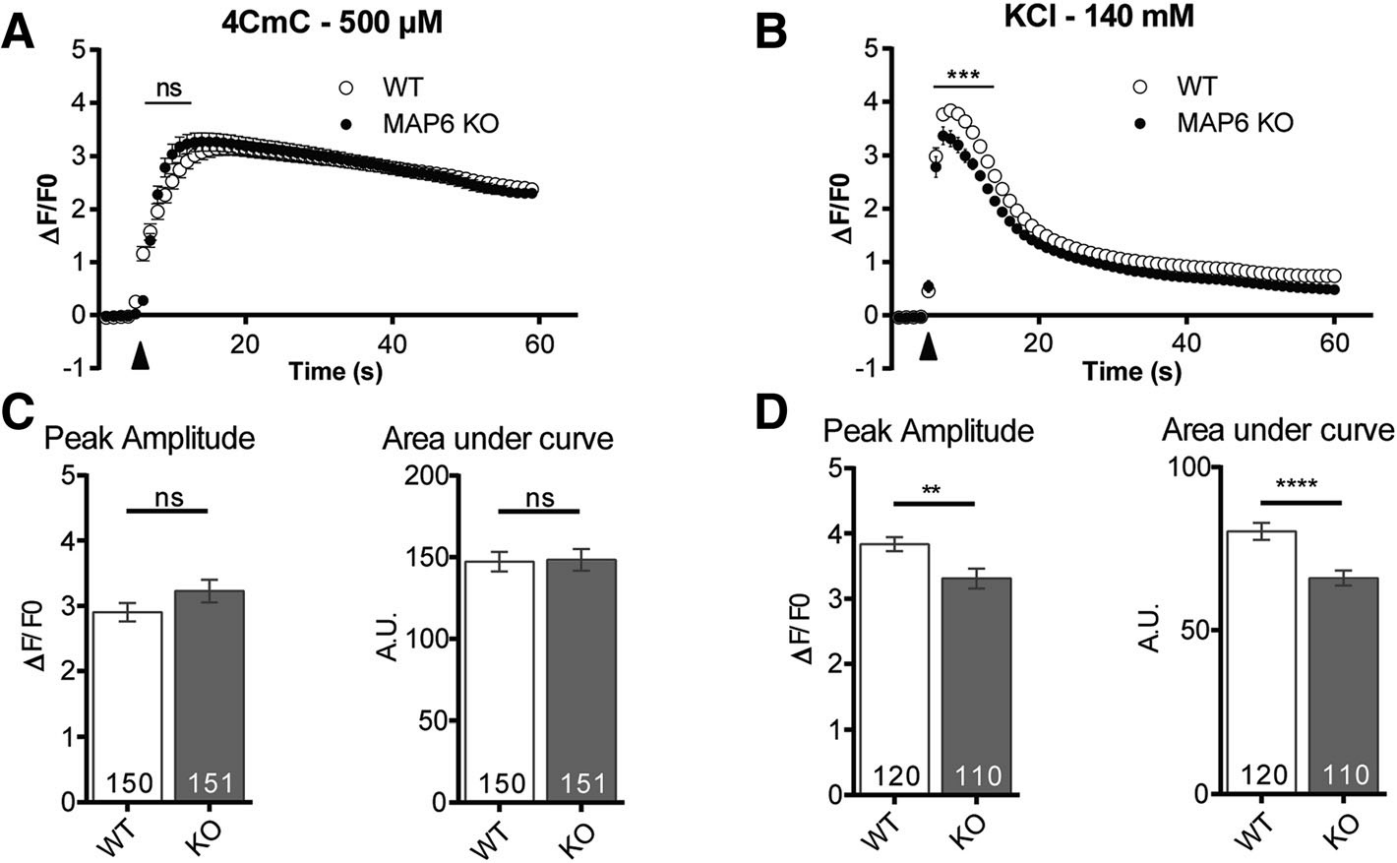
Calcium release is reduced in MAP6 KO myotubes compared to WT. **a** Direct stimulation of RyR1 by 500 μM 4CmC (black arrowhead). Fluorescence variations as a function of time are represented for WT myotubes (white dots) and MAP6 KO myotubes (black dots). Values are represented as means ± SEM for *n* = 150 and 151 myotubes respectively, from three different experiments, Student’s *t* test, ns: non-significant. **b** Membrane depolarization induced by addition of 140 mM KCl (black arrowhead). Fluorescence variations are presented for WT and MAP6 KO myotubes. Values are means ± SEM for *n* = 120 and 110 myotubes respectively, from three different experiments. Student’s *t* test, ***p < 0.001. **c**, **d** Maximal amplitude and area under curve for 4CmC stimulation (**c**) and KCl stimulation (**d**), with the number of analyzed-myotubes in each bar of the plot. Values are represented as means ± SEM. Student’s *t* test, ****p < 0.0001, **p < 0.01; ns: non-significant

Calcium release from the SR upon plasma membrane depolarization is a key step leading to muscle contraction and reduction in calcium release results in muscle weakness. Since alterations in muscle strength, in microtubules, and in SR organization have been observed in MAP6 KO muscles, calcium release was further analyzed in these muscles. Calcium imaging by confocal microscopy was performed on WT and MAP6 KO cultured myotubes. Two stimuli were used: a direct stimulation of the SR calcium channel, the ryanodine receptor (RyR1), by 500 μM 4-chloro-m-cresol (4CmC) (Fig. 6a, c) or a membrane depolarization using 140 mM KCl (Fig. 6b, d). This second stimulation protocol allows to assess the function of the whole calcium release complex (CRC) and the functional coupling between the voltage-activated calcium channel, the dihydropyridine receptor (DHPR), and RyR1. Only KCl-induced calcium release was significantly reduced in MAP6 KO myotubes compared to WT: both the peak amplitude and the area under the curve were reduced (Fig. 6d). The general organization of triads and microtubules was also studied in those WT and MAP6 KO cultured myotubes, and no gross modification was observed (Additional file 8: Figure S6). These results suggest that the coupling between RyR1 and DHPR, or the DHPR function, may be altered in MAP6 KO myotubes, whereas the RyR1 function and the SR calcium content were unmodified. Altogether, these results show that the muscle weakness of the MAP6 KO mice is related to a reduced calcium release.

## Discussion

Previous studies on the MAP6 KO mouse model were focused on neuronal function and shed light on numerous impairments [27, 28, 31], but no impact on skeletal muscle was demonstrated so far. The higher expression level of MAP6 in brain compared to skeletal muscle probably explains why the MAP6 KO mice have a major neurological phenotype. We have here characterized defects in muscle cell structure and function due to the absence of MAP6 proteins and shown that these animals experience muscle weakness with mild muscle atrophy. As muscle contraction was triggered in this study by a direct stimulation of muscle sarcolemma, it can be concluded that these mice have an intrinsic muscle dysfunction. We cannot exclude a small contribution of motor neuron dysfunction as the cause of the weakness; nevertheless, there was no sign of denervation. It is noteworthy that the observed reduction of the mechanical performance in MAP6 KO mice is comparable to that reported in other mouse models for congenital myopathies [54–56], arguing that the absence of MAP6 has a direct deleterious impact on muscle function.

The general structure of the MAP6 KO mice muscle fibers was not profoundly affected, but rearrangements in microtubules and SR organization were observed. A higher density of transverse microtubules was observed, suggesting a disorganization of the thick transverse bundles of microtubules. The EM analysis also revealed modifications of the SR which may be the result of the microtubule network disorganization, since both structures are in direct contact [57, 58]. Although subtle, these modifications of the subcellular structure of the muscle fibers may have important consequences. The stabilization of some microtubule bundles by another MAP (oMAP4) was for instance shown to play a role in muscle cell differentiation [8]. Modifications of SR structure have been observed in EDL muscles from calsequestrin-1 KO mice [59] or triadin-junctin double KO mice [52] and have been associated to defects in the contraction properties of the muscle fiber.

The consequences of MAP6 deletion on the skeletal muscle function were further analyzed by calcium imaging. Contraction of skeletal muscles is triggered by series of events leading to massive calcium efflux from SR cisternae via the intracellular RyR1 calcium channel. In MAP6 KO cells, the depolarization-induced calcium release, corresponding to the physiological activation, was reduced by 15%. As calcium release after direct RyR1 stimulation did not show any modification, this points to an altered DHPR-RyR1 coupling. This altered coupling is probably very transient because when observed in EM, both RyR1- and DHPR-containing membranes (respectively SR terminal cisternae and TT) were normally associated, and both proteins were normally expressed. Interestingly, it was demonstrated that MAP6 protein can stabilize microtubules against millimolar calcium concentration [60], a condition achieved only after muscle cell stimulation. It is therefore possible that the absence of MAP6 may alter the RyR1-DHPR crosstalk only during depolarization, when the cytosolic concentration of calcium rises, which would explain why no other variations are observed outside of muscle fiber stimulation. Recently, it was also shown that microtubule detyrosination could play an important role in muscle contraction efficiency by modulating the production of reactive oxygen species and calcium transient [3, 50, 51]. Although we could not demonstrate a change in the ratio of tyrosinated/detyrosinated microtubules, we cannot exclude that such a transitory modification of the microtubules during stimulation may affect muscle calcium release in MAP6 KO animals. The alterations observed in calcium release during excitation-contraction coupling are probably underlying the muscle weakness of the MAP6 KO mice.

The initial characterization of these mice showed several behavioral defects, among which a larger time spent standing still or walking at the expense of grooming and feeding [28]. Our demonstration that MAP6 KO mice also have a muscle weakness could explain in part this altered activity. Moreover, several abnormalities of the MAP6 KO mice were partially reverted using neuroleptics, leading to the proposal of this mouse line as a model for schizophrenia [28]. Interestingly, Chlorpromazine, a neuroleptic molecule used to revert MAP6 KO phenotype, was shown to increase the force in isolated muscle fibers at low concentration, by enhancing depolarization-induced calcium release [61]. A recent study also concluded to a correlative relationship between schizophrenia and muscle weakness in human patients [62]. It is therefore possible that together with a central nervous system dysfunction, skeletal muscle weakness contributes to the schizophrenia phenotype in the MAP6 KO mice.

## Conclusions

Although several studies showed major roles of MAPs in muscle cell differentiation and function [4, 8, 13], our report points for the first time to a defect in the contraction properties of adult muscle due to the absence of a MAP. The molecular bases for muscle weakness due to the absence of MAP6 probably rely on an alteration of the excitation-contraction coupling and calcium release, although we could not pinpoint a precise molecular mechanism so far. The subtle reorganization of the muscle fiber microtubule network could account for this excitation-contraction coupling alteration, but the involvement of other regulatory mechanisms could not be excluded as MAP6 proteins have been shown to interact with other proteins of the cytoskeleton like actin [23] or with Golgi elements [24, 25]. In conclusion, our work emphasizes that MAP6 deletion in mice leads to brain alterations as well as skeletal muscle defects that contributes to the mice schizophrenia-like phenotype.

## Additional files

Additional file 1: **Figure S1.** Multiples MAP6 isoforms are present in muscle cDNA. cDNA 5′-end amplification was performed from a primer in exon 2, which is common to all known MAP6 isoforms. The two major bands were extracted, purified, and partially sequenced allowing the identification of MAP6 transcripts (NCBI reference sequences MAP6-N: NM_010837.3, MAP6-E: NM_001048167.1, MAP6-F: NM_001043355.2). (JPG 853 kb)

Additional file 2: **Figure S2.** Dynamic and noninvasive investigation of *gastrocnemius* muscle bioenergetics using ^31^P-MRS. In vivo changes in *gastrocnemius* muscle PCr (A), ATP (B), and pH (C) throughout the 6-min fatiguing exercise and during the following 15-min recovery period. For each panel, the first time point (*t* = 0) indicates the basal value. Data are represented as means ± SEM for 6 WT and 7 MAP6 KO animals. Details on muscle bioenergetics are represented in Additional file 3: Table S1. (JPG 2119 kb)

Additional file 3: **Table S1.** *Gastrocnemius* muscle bioenergetics assessed in vivo using 31P-MRS. (DOCX 15 kb)

Additional file 4: **Figure S3.** Macroscopic organization of sarcomeres and triads are preserved in MAP6 KO fibers. FDB muscle fibers dissociated from WT and MAP6 KO adult mice were immuno-labeled with anti-α-actinin (green) and anti-RyR1 (red) antibodies for general appreciation of the fiber organization. Each image represents a single confocal plane. These images are representative from 6 to 10 randomly chosen fibers. Scale bar: 10 μm. (JPG 3236 kb)

Additional file 5: Figure S4. Total tubulin amount is not modified in MAP6 KO muscles. A) Representative western blot and B) quantitative analysis of β-tubulin amount in WT and MAP6 KO skeletal muscle homogenates. The amount of protein was normalized to GAPDH relative expression, and WT mean value set to 1. Values are represented as means ± SEM from *n* = 3 blots, Mann-Whitney test, ns: non-significant. (JPG 969 kb)

Additional file 6: **Figure S5.** The ratio between tyrosinated and detyrosinated microtubules seems unaffected in MAP6 KO. FDB muscle fibers dissociated from WT and MAP6 KO adult mice were labeled for tyrosinated tubulin (green) and detyrosinated tubulin (red). Each image represents a single confocal plane. Scale bars: 10 μm. The ratio between tyrosinated and detyrosinated microtubule network densities, reflecting respectively the dynamic and the stable microtubules, was measured depending on their orientation: either longitudinally oriented (a) or transversally oriented (b) compared to the fiber axis, on *n* = 14 WT and 20 MAP6 KO fibers. Values are represented as means ± SEM, Mann-Whitney test, ns: non-significant. (JPG 2421 kb)

Additional file 7: **Table S2.** Quantification of triads analyzed by EM. (DOCX 15 kb)

Additional file 8: **Figure S6.** Organization of triads and microtubules is similar in WT and MAP6 KO myotubes. WT and MAP6 primary cultures were differentiated for 3 days before being fixed and labeled with antibodies against triadin and RyR1 to visualize the triads and against tubulin to visualize the microtubules. No major difference is observed between the two genotypes for triadin, RyR, and tubulin. (JPG 1165 kb)

## Abbreviations

ATP: Adenosine triphosphate
B: Brain
C: C2C12 cell line
CRC: Calcium release complex
DHPR: Dihydropyridine receptor
EDL: Extensor digitorum longus
EM: Electron microscopy
FDB: Flexor digitorum brevis
GAPDH: Glyceraldehyde 3-phosphate dehydrogenase
H: Water control
KO: Knockout
M: Muscle
MAP: Microtubule-associated protein
MR: Magnetic resonance
PCr: Phosphocreatine
RyR: Ryanodine receptor
SERCA: Sarco-endoplasmic reticulum Ca^2+^ ATPase
SR: Sarcoplasmic reticulum
STOP: Stable tubule-only polypeptide
TT: Transverse tubule
WT: Wildtype
