# Supplementary figures and images for: Deletion of the microtubule-associated protein 6 (MAP6) results in skeletal muscle dysfunction
Source: Skelet Muscle. 2018 Sep 19;8:30. doi: 10.1186/s13395-018-0176-8 (PMC6147105; doi:10.1186/s13395-018-0176-8)

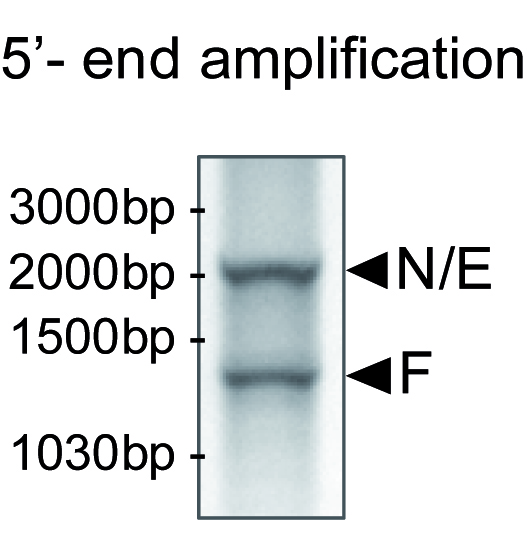

Supplement: Supplementary file 1 — Figure S1. Multiples MAP6 isoforms are present in muscle cDNA. cDNA 5′-end amplification was performed from a primer in exon 2, which is common to all known MAP6 isoforms. The two major bands were extracted, purified, and partially sequenced allowing the identification of MAP6 transcripts (NCBI reference sequences MAP6-N: NM_010837.3, MAP6-E: NM_001048167.1, MAP6-F: NM_001043355.2). (JPG 853 kb) [file 13395_2018_176_MOESM1_ESM.jpg]

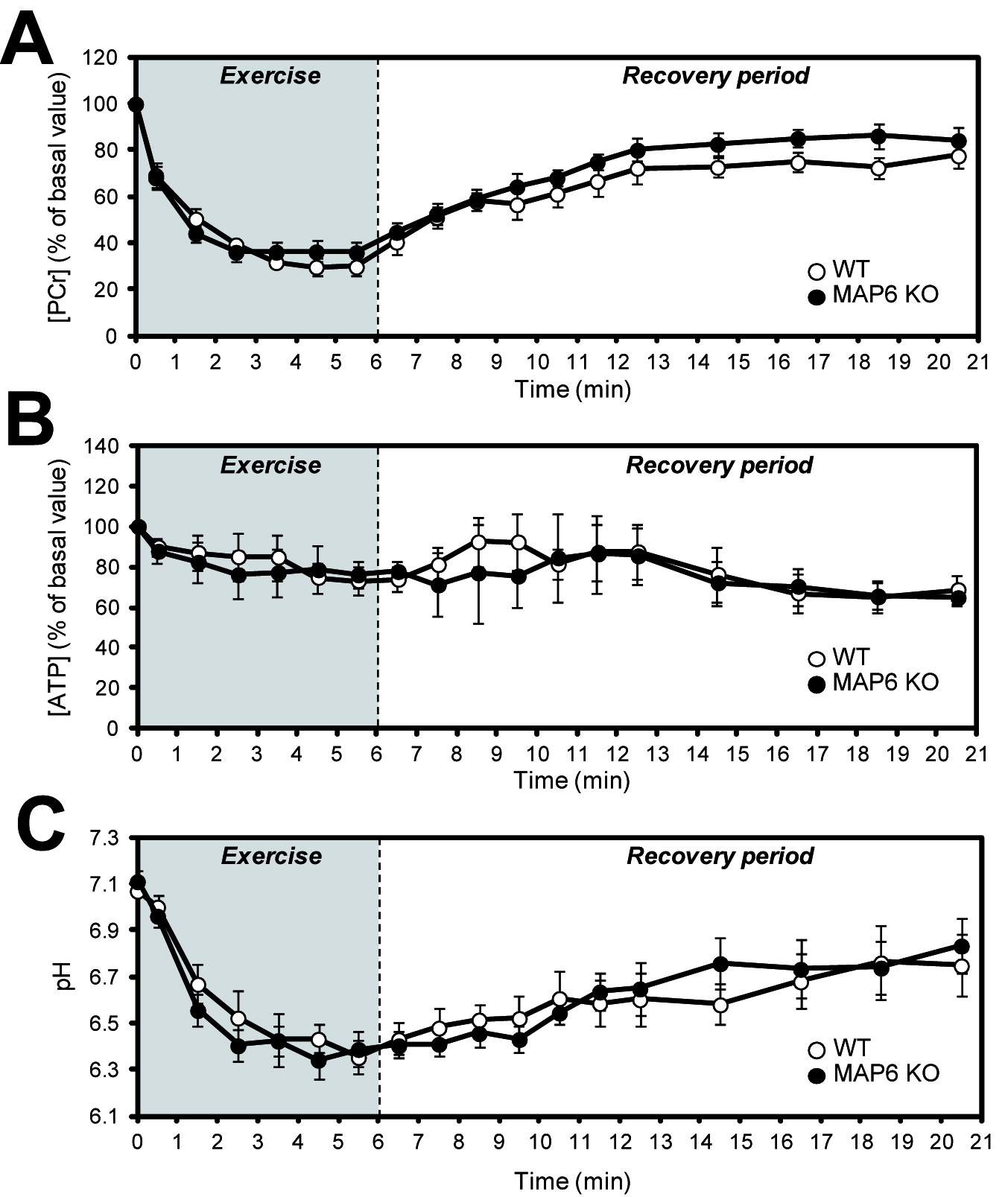

Supplement: Supplementary file 2 — Figure S2. Dynamic and noninvasive investigation of gastrocnemius muscle bioenergetics using 31P-MRS. In vivo changes in gastrocnemius muscle PCr (A), ATP (B), and pH (C) throughout the 6-min fatiguing exercise and during the following 15-min recovery period. For each panel, the first time point (t = 0) indicates the basal value. Data are represented as means ± SEM for 6 WT and 7 MAP6 KO animals. Details on muscle bioenergetics are represented in Additional file 3: Table S1. (JPG 2119 kb) [file 13395_2018_176_MOESM2_ESM.jpg]

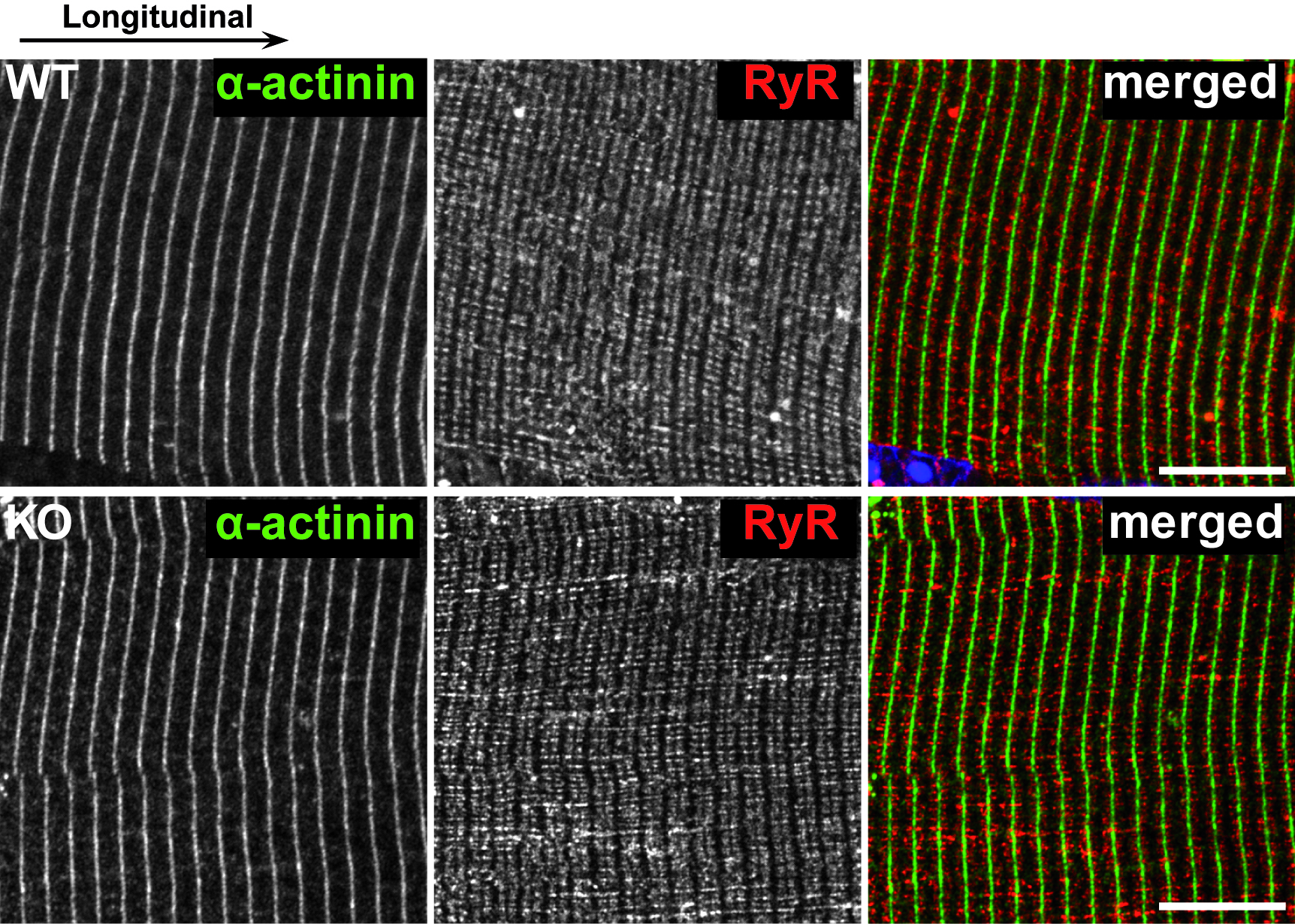

Supplement: Supplementary file 4 — Figure S3. Macroscopic organization of sarcomeres and triads are preserved in MAP6 KO fibers. FDB muscle fibers dissociated from WT and MAP6 KO adult mice were immuno-labeled with anti-α-actinin (green) and anti-RyR1 (red) antibodies for general appreciation of the fiber organization. Each image represents a single confocal plane. These images are representative from 6 to 10 randomly chosen fibers. Scale bar: 10 μm. (JPG 3236 kb) [file 13395_2018_176_MOESM4_ESM.jpg]

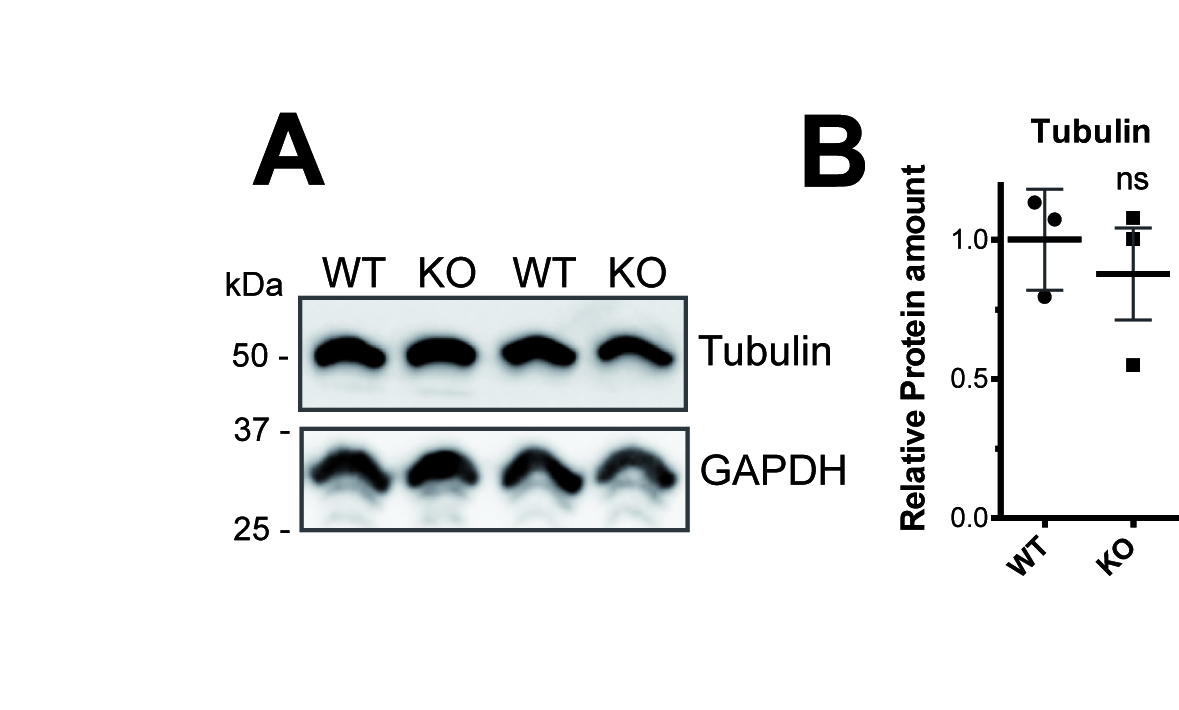

Supplement: Supplementary file 5 — Figure S4. Total tubulin amount is not modified in MAP6 KO muscles. A) Representative western blot and B) quantitative analysis of β-tubulin amount in WT and MAP6 KO skeletal muscle homogenates. The amount of protein was normalized to GAPDH relative expression, and WT mean value set to 1. Values are represented as means ± SEM from n = 3 blots, Mann-Whitney test, ns: non-significant. (JPG 969 kb) [file 13395_2018_176_MOESM5_ESM.jpg]

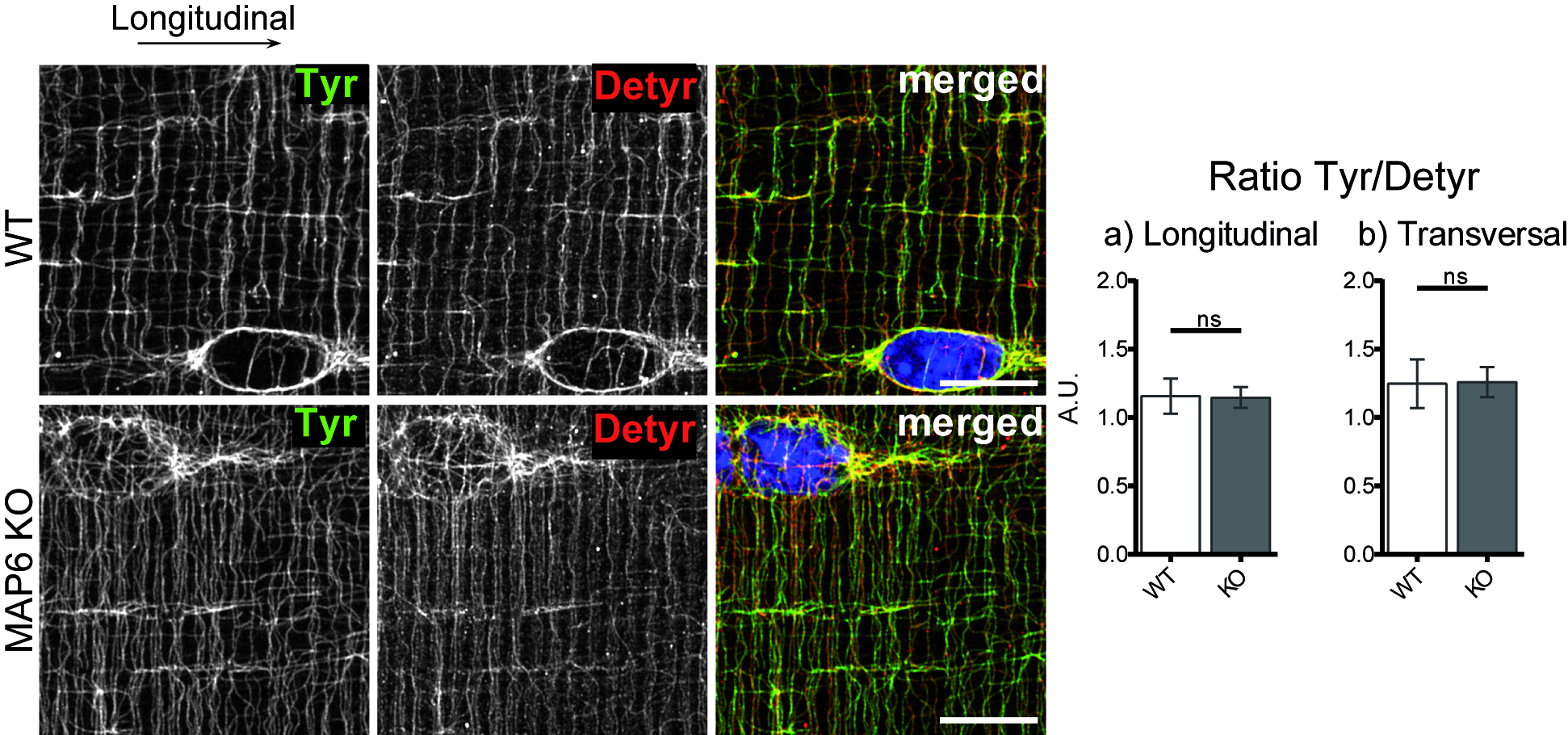

Supplement: Supplementary file 6 — Figure S5. The ratio between tyrosinated and detyrosinated microtubules seems unaffected in MAP6 KO. FDB muscle fibers dissociated from WT and MAP6 KO adult mice were labeled for tyrosinated tubulin (green) and detyrosinated tubulin (red). Each image represents a single confocal plane. Scale bars: 10 μm. The ratio between tyrosinated and detyrosinated microtubule network densities, reflecting respectively the dynamic and the stable microtubules, was measured depending on their orientation: either longitudinally oriented (a) or transversally oriented (b) compared to the fiber axis, on n = 14 WT and 20 MAP6 KO fibers. Values are represented as means ± SEM, Mann-Whitney test, ns: non-significant. (JPG 2421 kb) [file 13395_2018_176_MOESM6_ESM.jpg]

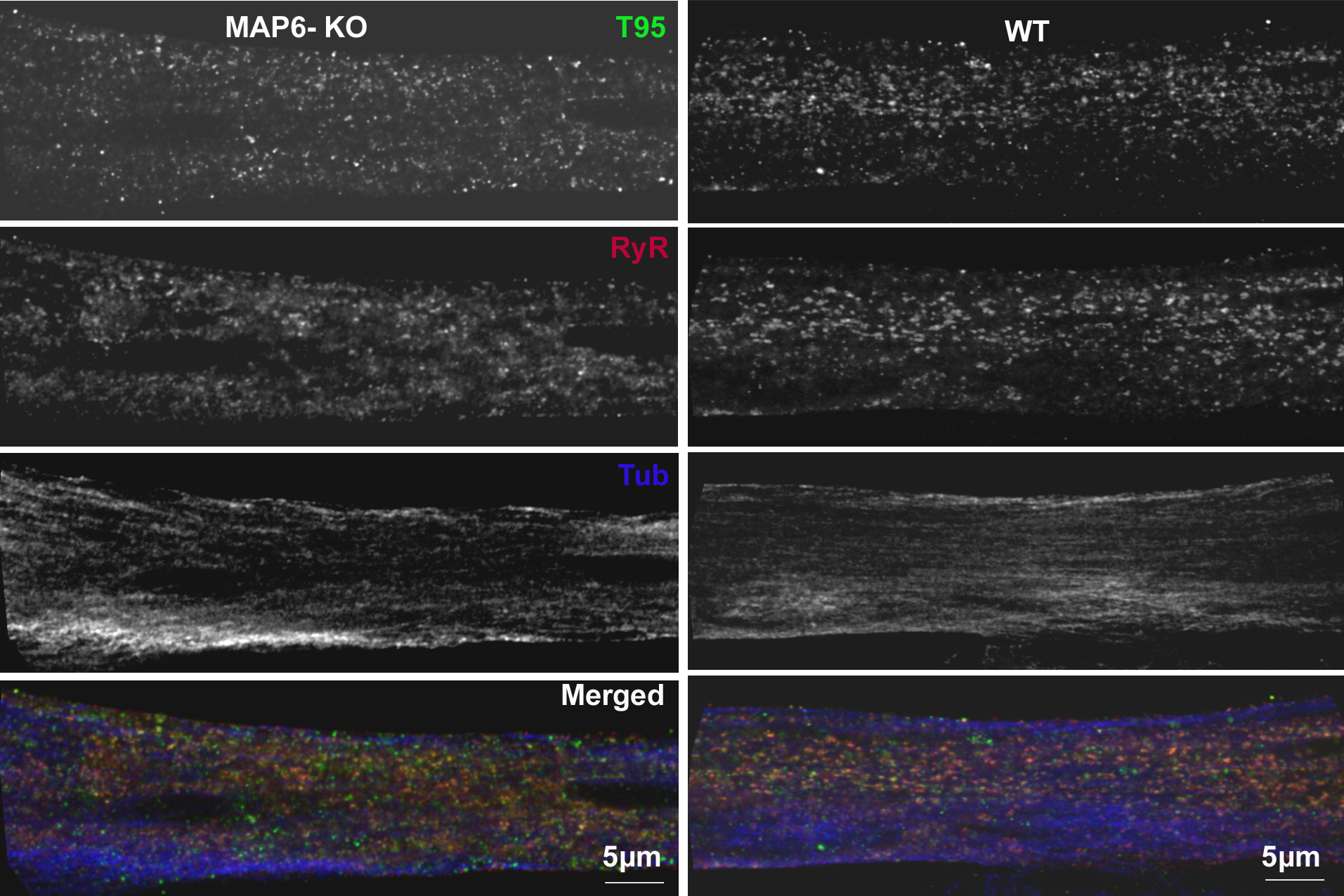

Supplement: Supplementary file 8 — Figure S6. Organization of triads and microtubules is similar in WT and MAP6 KO myotubes. WT and MAP6 primary cultures were differentiated for 3 days before being fixed and labeled with antibodies against triadin and RyR1 to visualize the triads and against tubulin to visualize the microtubules. No major difference is observed between the two genotypes for triadin, RyR, and tubulin. (JPG 1165 kb) [file 13395_2018_176_MOESM8_ESM.jpg]
