# Supplementary material for: Deletion of the microtubule-associated protein 6 (MAP6) results in skeletal muscle dysfunction
Source: Skelet Muscle. 2018 Sep 19;8:30. doi: 10.1186/s13395-018-0176-8 (PMC6147105; doi:10.1186/s13395-018-0176-8)
Supplement: Supplementary file 3 — Table S1. Gastrocnemius muscle bioenergetics assessed in vivo using 31P-MRS. (DOCX 15 kb) [file 13395_2018_176_MOESM3_ESM.docx]

**Table S1: *Gastrocnemius* muscle bioenergetics assessed in vivo using ^31^P-MRS**

|  | WT | MAP6 KO |
| --- | --- | --- |
| Basal [PCr]/[ATP] | 2.1 ± 0.2 | 1.8 ± 0.2 |
| Basal pH | 7.07 ± 0.02 | 7.11 ± 0.04 |
| τPCr_ex_ (min) | 1.4 ± 0.4 | 0.8 ± 0.2 |
| End exercise PCr level (% of basal) | 30 ± 4 | 36 ± 4 |
| End exercise ATP level (% of basal) | 73 ± 7 | 76 ± 7 |
| End exercise pH | 6.36 ± 0.07 | 6.39 ± 0.07 |
| τPCr_rec_ (min) | 3.2 ± 0.5 | 4.2 ± 0.5 |

τPCr_ex_, time constant of PCr degradation at the exercise start; τPCr_rec_, time constant of PCr re-synthesis at the start of the post-exercise recovery period Values are means ± SEM, for 6 WT and 7 MAP6 KO animals, Mann-Whitney tests, no significant differences between WT and MAP6 KO.
