# Supplementary material for: Deletion of the microtubule-associated protein 6 (MAP6) results in skeletal muscle dysfunction
Source: Skelet Muscle. 2018 Sep 19;8:30. doi: 10.1186/s13395-018-0176-8 (PMC6147105; doi:10.1186/s13395-018-0176-8)
Supplement: Supplementary file 7 — Table S2. Quantification of triads analyzed by EM. (DOCX 15 kb) [file 13395_2018_176_MOESM7_ESM.docx]

**Table S2: Quantification of triads analyzed by EM**

|  |  | WT | MAP6 KO |
| --- | --- | --- | --- |
| A | No. of triads /100 µm^2^ | 74.4 ± 1.5 | 75.5 ± 1.3 |
| B | Longitudinal triads (%) | 0.9 ± 0.3 | 0.8 ± 0.3 |
| C | **Oblique triads (%)** | **1.7 ± 0.4** | **5.6 ± 0.8 *** |

Data are shown as mean ± SEM. *p< 0.01 vs WT. Student t test

Sample size: 20-30 fibers, from 3 WT and 3 MAP6 KO, and 6 micrographs/fiber (14k magnification images in longitudinal sections).
